# Vitamin C in plasma is inversely related to blood pressure and change in blood pressure during the previous year in young Black and White women

**DOI:** 10.1186/1475-2891-7-35

**Published:** 2008-12-17

**Authors:** Gladys Block, Christopher D Jensen, Edward P Norkus, Mark Hudes, Patricia B Crawford

**Affiliations:** 1 School of Public Health, 50 University Hall, University of California, Berkeley, CA 94720, USA; 2 Department of Medical Research, Montefiore Medical Center North Division, 600 East 233Street, Bronx, NY 10466, USA; 3 Nutritional Sciences and Toxicology, 119 Morgan Hall, University of California, Berkeley, CA 94720, USA

## Abstract

**Background:**

The prevalence of hypertension and its contribution to cardiovascular disease risk makes it imperative to identify factors that may help prevent this disorder. Extensive biological and biochemical data suggest that plasma ascorbic acid may be such a factor. In this study we examined the association between plasma ascorbic acid concentration and blood pressure (BP) in young-adult women.

**Methods:**

Participants were 242 Black and White women aged 18–21 yr from the Richmond, CA, cohort of the National Heart, Lung and Blood Institute Growth and Health Study. We examined the associations of plasma ascorbic acid with BP at follow-up year 10, and with change in BP during the previous year.

**Results:**

In cross-sectional analysis, plasma ascorbic acid at year 10 was inversely associated with systolic BP and diastolic BP after adjusting for race, body mass index, education, and dietary intake of fat and sodium. Persons in the highest one-fourth of the plasma ascorbic acid distribution had 4.66 mmHg lower systolic BP (95% CI 1.10 to 8.22 mmHg, p = 0.005) and 6.04 mmHg lower diastolic BP (95% CI 2.70 to 9.38 mmHg, p = 0.0002) than those in the lowest one-fourth of the distribution. In analysis of the *change *in BP, plasma ascorbic acid was also inversely associated with change in systolic BP and diastolic BP during the previous year. While diastolic blood pressure among persons in the lowest quartile of plasma ascorbic acid increased by 5.97 mmHg (95% CI 3.82 to 8.13 mmHg) from year 9 to year 10, those in the highest quartile of plasma vitamin C increased by only 0.23 mmHg (95% CI -1.90 to +2.36 mmHg) (test for linear trend: p < 0.0001). A similar effect was seen for change in systolic BP, p = 0.005.

**Conclusion:**

Plasma ascorbic acid was found to be inversely associated with BP and change in BP during the prior year. The findings suggest the possibility that vitamin C may influence BP in healthy young adults. Since lower BP in young adulthood may lead to lower BP and decreased incidence of age-associated vascular events in older adults, further investigation of treatment effects of vitamin C on BP regulation in young adults is warranted.

## Background

Elevated blood pressure (BP) is a major public health problem in the United States with almost 60% of American adults having pre-hypertension or hypertension [1]. The disorder is prevalent in all major ethnic groups, although Blacks are disproportionately affected [1]. As elevated BP is a strong and independent risk factor for cardiovascular and renal disease morbidity and mortality [2], identifying preventive measures is an important public health goal.

The Dietary Approaches to Stop Hypertension (DASH) trial demonstrated that diet is an important factor in BP regulation. In that study, a diet rich in vegetables, fruit, low-fat dairy foods, and with reduced saturated and total fat intake significantly reduced systolic BP and diastolic BP over a period of 8 weeks compared to a control diet [3]. DASH diet constituents believed to contribute to the observed BP-lowering effect include calcium, magnesium, potassium, dietary fiber, and the reduced fat content of the diet [3,4]. The DASH diet is also rich in vitamin C, another possible contributor to the observed BP effect [5]. Mechanistic studies have demonstrated a role of vitamin C in maintaining the normal production and biological activity of endothelium-derived nitric oxide which plays a role in vascular tone and reactivity [6,7]. Thus, plasma ascorbic acid concentration should be investigated for its possible role in lowering or maintaining normal BP.

Numerous cross-sectional studies have observed highly significant inverse relationships between BP and circulating ascorbic acid concentration [8-18]. Almost all such studies have focused on populations that are middle-aged and older, and intervention research has typically focused on treatment of those with already elevated blood pressure. However, we hypothesized that an ascorbic acid association in a young normotensive population might provide clues to the primary prevention of hypertension. Examining this relationship in young adults is potentially important because maintaining healthy BP in healthy young adults may lead to a lower BP in older adults, and consequently a lower incidence of age-associated vascular events [2]. Therefore, we investigated this relationship among 18–21 year old Black and White women enrolled in the National Heart, Lung, and Blood Institute (NHLBI) Growth and Health Study. We hypothesized that plasma ascorbic acid concentration at the 10th annual follow-up visit would be inversely associated with BP, and inversely associated with change in BP during the previous year, in this population group.

## Methods

### Participants

In 1985, the NHLBI initiated the Growth and Health Study, a 10-year longitudinal multicenter study to investigate the development of obesity in Black and White girls during adolescence, along with its environmental, psychosocial, and cardiovascular disease risk factor correlates. Black and White girls, ages 8 to 11 years, were recruited from Richmond, CA, Cincinnati, OH, and Washington, DC areas. Institutional review board approval was obtained at all participating centers. The parents or guardians of participants gave informed consent for the children to participate. Girls were followed annually through ages 18 to 21 years, with a follow-up rate of 90% at the 10^th ^annual follow-up visit. The sample design, recruitment and selection procedures used for the Growth and Health Study have been previously described [19]. For this investigation, plasma ascorbic acid samples were obtained only from girls in the Richmond, CA cohort at the 10^th ^annual visit. The blood draw for plasma ascorbic acid was implemented midway through the annual data collection period and a total of 335 participants were seen in the clinic after plasma sample collection was initiated. Plasma samples were successfully obtained from 242 participants (72%), consisting of 155 Blacks and 87 Whites. The 93 who did not participate in the extra blood draw were either seen on a day when the ascorbic acid study staff was not present to take their blood, were pregnant, or refused the extra procedure.

### Data collection

#### BP

BP measurements were obtained by trained examiners according to a standard protocol [20]. Participants were seated with feet resting flat on a surface and the right arm resting at heart level. Participants were seated for at least 5 minutes before BP measurements were begun. The appropriate cuff was selected from 5 cuff sizes and placed around the upper arm. Using a standard mercury sphygmomanometer (Baum Desktop model with V-Lok cuffs), three consecutive readings were taken using the same arm and waiting at least 30 seconds between readings. The second and third readings were averaged to determine the mean systolic BP, defined as the onset of the first two consecutive beats (first Korotkoff phase), and the mean diastolic BP, defined as the point at which the sound disappeared (fifth Korotkoff phase). BP was obtained at both the 9th and 10^th ^annual visits.

#### Plasma ascorbic acid

Participants were instructed to fast for at least 12 hours before the blood draw. Plasma ascorbic acid was determined using the 2,4-dinitrophenylhydrazine method [21], which has been shown to correlate well with HPLC methods [22]. Plasma was stabilized with 10% metaphosphoric acid, frozen at -70°C within 4 hours of the collection, and analyzed within six months. Plasma ascorbic acid was assayed only at the 10^th ^annual visit. Blood stored from earlier visits could not be used for assaying ascorbic acid because it had not been stabilized with metaphosphoric acid.

#### Anthropometry

At the 10^th ^annual visit, participants were weighed using an electronic scale (SECA Alpha 770) in gowns without shoes. Height was measured using a custom portable stadiometer (Creative Health Products, Plymouth, MI). Participants wore socks and were positioned with heels together, toes apart at a 45 degree angle, and head in the Frankfort horizontal plane. Both weight and height were measured twice and repeated a third time if values differed by more than 0.3 kg and 0.5 cm, respectively. Body mass index (BMI) was calculated as kg/m^2^.

#### Socioeconomic and lifestyle variables

Information on socioeconomic and lifestyle variables was ascertained by questionnaire at the 10^th ^annual visit. Smoking was assessed by asking how many days participants smoked in the past 30 days and how many cigarettes were smoked per day. The number of smoking days/month was then multiplied by the number of cigarettes/day to create a continuous variable, cigarettes smoked/month. Alcohol consumption was assessed in a similar fashion, with 1 drink being equivalent to 12 oz. (355 mL) of beer or 6 oz. (177 mL) of wine. The number of hours/week of television or videos watched was assessed as a proxy for physical inactivity. Pregnancy history and current contraceptive use was also obtained.

#### Dietary variables

Dietary information at the 10^th ^annual visit was determined using 3-day dietary records, covering two consecutive weekdays and one weekend day. Participants used rulers, pictures, and thickness guides to measure and quantify the amount of food consumed. Dietary records were reviewed with a dietitian and analyzed for nutrient composition by the Cincinnati Dietary Center using the University of Minnesota Nutrition Coordinating Center database. The method used for estimating nutrient intake had been previously validated in this population [23]. All micronutrient intake estimates include amounts provided by supplements. Estimates of dietary sodium intake were from food only and no attempt was made to collect information on added dietary salt intake.

### Statistical analyses

Unpaired two-tailed t-tests and Wilcoxon two-sample tests were used to compare baseline characteristics of Black and White study participants. Plasma ascorbic acid at year 10 was divided into quartiles, and least squares mean diastolic and systolic BP, adjusted for race, BMI, education, and intake of fat and sodium, were calculated for each quartile using analysis of covariance. Change in BP was calculated as the systolic and diastolic values at year 10 minus their respective values at year 9, and adjusted changes were calculated using analysis of covariance; year 9 BP was included as a covariate in the model. The relationships between plasma ascorbic acid and BP at the 10^th ^annual visit, as well as between plasma ascorbic acid at year 10 and change in BP in the previous year, were also examined as continuous variables using multivariable linear regression models. The interaction of race and BMI with the independent variable was assessed using cross-product terms in the regression models. Annual household income and education were highly correlated. However, education explained more of the variance, and consequently education was used in the models. A number of other covariates were considered as potential confounders, including intake of total energy, potassium, calcium, dietary antioxidants, and fiber, pregnancy history, use of birth control pills, smoking, alcohol consumption, and number of hours/week of television or video watching. However, none was statistically significant and these variables were not included in the final models. Statistical analyses were conducted with SAS 9.1.

## Results

Descriptive characteristics and comparisons of the total sample and by race are presented in Table 1. Mean diastolic BP, age, weight, BMI, energy intake, and plasma ascorbic acid concentrations did not differ significantly by race. Black women had higher mean systolic BP, watched more hours/week of television or videos, consumed more fat/day, and lived in households with lower incomes as compared to White women. Blacks also smoked significantly fewer cigarettes, drank less alcohol, and were more likely to have been pregnant (data not shown.) However, race did not significantly modify the relationship between plasma ascorbic acid and BP (interaction terms for systolic and diastolic BP were p = 0.42 and p = 0.47, respectively). Therefore, statistical models included all 242 subjects and were not stratified by race.

**Table 1:** Characteristics of 242 young-adult women in the Richmond, CA cohort of the NHLBI Growth and Health Study, 1996–1997.

	**All** **(n = 242)**		**Blacks** **(n = 155)**		**Whites** **(n = 87)**		
	**Mean**	**SD**	**Mean**	**SD**	**Mean**	**SD**	p*

**Systolic BP (mmHg)**	109.8	10.3	111.1	10.8	107.4	9.0	0.007
**Diastolic BP (mmHg)**	66.9	9.0	67.4	9.0	66.1	9.0	0.31
**Plasma vitamin C (mg/dL)^†^**	1.2	0.5	1.2	0.4	1.2	0.6	0.34
**Age (yr)**	19.1	0.6	19.2	0.7	19.1	0.6	0.28
**Weight (kg)**	70.9	19.3	71.2	19.3	70.4	19.3	0.74
**BMI (kg/m^2^)**	26.1	6.7	26.4	6.6	25.7	6.9	0.40
**Television or video watching (hr/wk)**	32.0	24.1	39.3	23.9	19.0	18.4	< 0.0001
**Household income ($)^‡^**	20,000–29,999		10,000–19,999		30,000–39,999		< 0.0001
**Total energy intake (kcal/d)**	1919.6	787.2	1985.8	827.7	1809.9	706.4	0.11
**Total fat intake (gm/d)**	74.1	36.8	79.3	36.2	65.5	36.2	0.006
Dietary vitamin C intake (mg/d)** Mean (median, 25^th ^75^th ^%ile)	121.7 (79.3)	176.6 (38.9, 128.2)	128.1 (80.0)	203.4 (43.4, 126.1)	111.1 (63.5)	120.3 (31.0, 136.5)	0.43

* P-values pertain to comparison between Blacks and Whites.

^† ^Multiply by 56.8 to convert to SI units, μmol/L

‡ Household income values are the categories in which the median fell.

** Values in parentheses represent median (in the Means column) and 25^th^/75^th ^percentiles (in the Standard Deviations column)

For comparison of BP in categories of plasma ascorbic acid, the plasma ascorbic acid distribution among participants at visit 10 was divided into fourths. Mean concentrations of ascorbic acid, by quartile, were as follows: Quartile 1: 0.59 mg/dL (SD 0.18 mg/dL); Quartile 2: 1.03 mg/dL (SD 0.10 mg/dL); Quartile 3: 1.36 mg/dL (SD 0.10 mg/dL); and Quartile 4: 1.83 mg/dL (SD 0.34 mg/dL). (Means in SI units: 33.51 μmol/L, 58.50 μmol/L, 77.25 μmol/L, 103.94 μmol/L for quartiles 1 through 4, respectively.)

**Figure 1:**
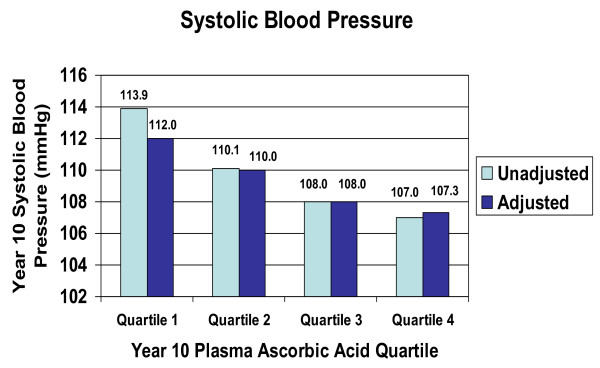
Systolic blood pressure by quartiles of plasma ascorbic acid, NHLBI Growth and Health Study, Richmond, CA cohort, 1996–1997 (tests for linear trend: unadjusted p = 0.0001; adjusted for race, BMI, education, dietary fat, dietary sodium, p = 0.005). Plasma ascorbic acid concentrations by quartile: Q1, 0.59 mg/dL (SD 0.18); Q2, 1.03 mg/dL (SD 0.10); Q3, 1.36 mg/dL (SD 0.10); Q4, 1.83 mg/dL (SD 0.34). (SI units: 33.51 μmol/L, 58.50 μmol/L, 77.25 μmol/L, 103.94 μmol/L)

**Figure 2:**
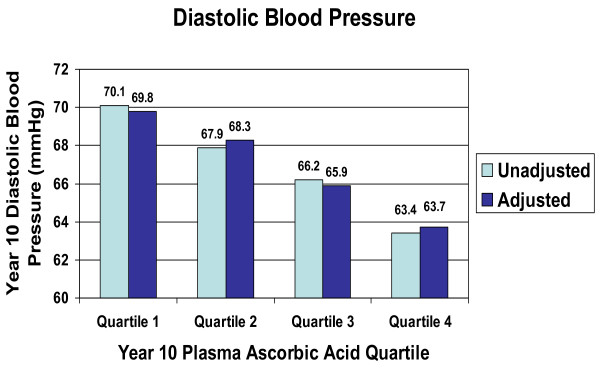
Diastolic blood pressure by quartiles of plasma ascorbic acid, NHLBI Growth and Health Study, Richmond, CA cohort, 1996–1997 (tests for linear trend: unadjusted p = 0.0001; adjusted for race, BMI, education, dietary fat, dietary sodium, p = 0.0002). Plasma ascorbic acid concentrations by quartile: Q1, 0.59 mg/dL (SD 0.18); Q2, 1.03 mg/dL (SD 0.10); Q3, 1.36 mg/dL (SD 0.10); Q4, 1.83 mg/dL (SD 0.34). (SI units: 33.51 μmol/L, 58.50 μmol/L, 77.25 μmol/L, 103.94 μmol/L)

In cross-sectional analyses, we examined BP across quartiles of plasma ascorbic acid at the 10^th ^annual visit. There was a significant inverse linear trend in both systolic BP (Figure 1) and diastolic BP (Figure 2) across the quartiles of plasma ascorbic acid concentration (unadjusted p = 0.0001, respectively). In regression analysis using the quartiles as predictor variables, after adjustment for BMI, race, education, and dietary fat and sodium intake, these inverse linear trends remained significant for both systolic BP and diastolic BP, with substantial differences in BP across the quartiles. Persons in the upper one-fourth of the plasma ascorbic acid distribution had 4.66 mmHg lower systolic BP (95% CI 1.10 to 8.22 mmHg, p = 0.005) than those in the lower one-fourth of the plasma ascorbic acid distribution. For diastolic BP, persons in the upper one-fourth of the plasma ascorbic acid distribution had 6.04 mmHg lower diastolic BP (95% CI 2.70 to 9.38 mmHg, p = 0.0002) than those in the lower quartile of ascorbic acid. We also performed multiple regression analysis of ascorbic acid as a continuous variable, with BP as the dependent variable, plasma ascorbic acid as the primary predictive variable, and adjusting for BMI, race, education, dietary fat and sodium intake. Cross-sectionally, as plasma ascorbic acid concentration increased by 1 mg/dL (56.8 μmol/L), systolic BP decreased by 4.1 mmHg (95% CI -6.6 to -1.7 mmHg) and diastolic BP decreased by 4.0 mmHg (95% -6.4 to -1.7 mmHg).

**Figure 3:**
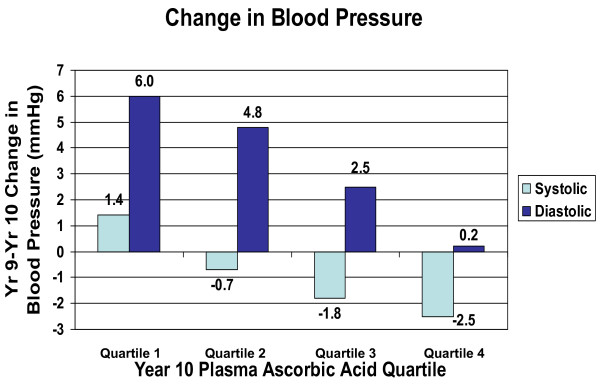
Change in blood pressure between visit 9 and visit 10, by quartiles of plasma ascorbic acid at visit 10, NHLBI Growth and Health Study, Richmond, CA cohort, 1996–1997 (tests for linear trend: systolic BP p = 0.005; diastolic BP p < 0.0001; adjusted for race, BMI, education, dietary fat, dietary sodium). Plasma ascorbic acid concentrations by quartile: Q1, 0.59 mg/dL (SD 0.18); Q2, 1.03 mg/dL (SD 0.10); Q3, 1.36 mg/dL (SD 0.10); Q4, 1.83 mg/dL (SD 0.34). (SI units: 33.51 μmol/L, 58.50 μmol/L, 77.25 μmol/L, 103.94 μmol/L)

We also examined the association between plasma ascorbic acid concentration at year 10 and *change *in BP between years 9 and 10. Change in both systolic BP and diastolic BP was inversely associated with plasma ascorbic acid concentration at year 10 (Figure 3) after adjustment for BMI, race, education, and dietary fat and sodium intake. Systolic BP increased by 1.40 mmHg (95% CI -0.76 to +3.57 mmHg) among those in the lowest quartile of the plasma ascorbic acid distribution, but decreased in persons in higher quartiles of plasma ascorbic acid (test for linear trend in change in BP across quartiles of year 10 ascorbic acid: p = 0.005). Diastolic BP increased overall, but the increase was less with increasing quartiles of plasma ascorbic acid. While diastolic blood pressure among persons in the lowest quartile of plasma ascorbic acid increased by 5.97 mmHg (95% CI 3.82 to 8.13 mmHg) from year 9 to year 10, those in the highest quartile of plasma vitamin C increased by only 0.23 mmHg (95% CI -1.90 to +2.36 mmHg) (test for linear trend: p < 0.0001). Control for weight change between years 9 and 10 made essentially no difference in these results (data not shown).

## Discussion

We found plasma ascorbic acid concentration to be inversely associated with systolic and diastolic BP in young-adult Black and White women, independent of race, BMI, education, diet, and lifestyle factors. A 1 mg/dL (56.8 μmol/L) increase in plasma ascorbic acid levels was associated with 4.1 mmHg lower systolic blood pressure and 4.0 mmHg lower diastolic blood pressure. Plasma ascorbic acid levels in this cohort ranged from 0.22 to 3.13 mg/dL (12.5 to 177.8 μmol/L), indicating that a 1 mg/dL change in plasma ascorbic acid level is achievable in this young population.

Plasma ascorbic acid concentration was also inversely and significantly associated with *change *in BP during the previous year. It is particularly notable that there was very little increase in diastolic BP in persons in the highest quartile of ascorbic acid.

The magnitude of the decrease in systolic and diastolic blood pressure associated with plasma ascorbic acid is consistent with the range of values found in other cross-sectional studies [8-18]. A review by Ness [18] found that the blood pressure difference associated with a 50 μmol/L (0.88 mg/dL) difference in ascorbic acid concentration ranged from -2.6 to -9.4 mmHg for diastolic blood pressure, and from -3.6 to -17.8 mmHg for systolic blood pressure.

Intervention trials have been less consistent [24-37]. Many of the studies that failed to find a treatment effect have had design limitations such as no control group or no placebo control, short duration, small sample size, concomitant use of non-study dietary supplements containing vitamin C, and varying or inadequately described methods for measuring BP; or they were post-hoc analyses where change in BP was not a primary endpoint of the trial. On the other hand, some studies have found significant reductions in BP as a result of treatment with vitamin C [38-41].

The DASH study [3] is the major intervention trial of nutritional factors, and our results are comparable in magnitude to those of DASH. Among all participants in the DASH study, the DASH diet lowered mean systolic BP by 5.5 mmHg, and lowered mean diastolic BP by 3.0 mmHg. In the present study, the difference between the mean BP in the first and fourth quartiles of ascorbic acid concentration was 4.7 mmHg systolic, and 6.1 mmHg diastolic BP. This suggests the possibility that if the ascorbic acid concentration of those in the lower one-fourth of the distribution could be increased to the ascorbic acid concentration of those in the top one-fourth of the distribution, an effect comparable to that of the DASH diet could be achieved. The present study was not an intervention study and such an intervention effect is only a hypothesis, but one which would seem to merit follow-up.

The ranges of plasma ascorbic acid seen in our sample are very consistent with those found in the United States. We examined data from the National Health and Nutrition Examination Survey (NHANES) 2003–2004, among women in the same age group, 18–21 years. The mean plasma ascorbic acid concentrations in the four quartiles were 0.43 mg/dL, 0.84 mg/dL, 1.14 mg/dL, and 1.59 mg/dL (24.1, 47.8, 64.8, 90.1 μmol/L). (Block, unpublished data.) In our sample those means were 0.59, 1.03, 1.36 and 1.83 mg/dL. Thus, in the present sample the first three quartiles were similar although slightly higher than those seen in representative national data. The lower two quartiles represent ascorbic acid concentrations most likely obtained from fruits, vegetables and fortified foods such as cereals. A diet rich in fruits and vegetables can produce a concentration similar to that seen in the third quartile. The concentration in the fourth quartile represents a level in which supplements containing vitamin C have made a substantial contribution.

There is substantial biologic rationale for a causal role of ascorbic acid in maintaining normal BP. Numerous oxidative mechanisms appear to be involved in the pathogenesis of hypertension [6,7,42]. One well-studied oxidative stress biomarker is F_2_-isoprostane, a vasoactive product of lipid peroxidation [43]; we have shown in a randomized trial that supplementation with vitamin C, but not with vitamin E, significantly reduces F_2_-isoprostane [44]. Similarly inflammation, consistently associated with increased risk of cardiovascular disease, is associated with impaired endothelium-dependent vasodilation and hypertension [45]. Bautista found a progressive increase in blood pressure with increases in high-sensitivity C-reactive protein (hsCRP), a sensitive marker of inflammation [45]. We showed, in a randomized trial, that vitamin C but not vitamin E significantly lowered hsCRP, to an extent equivalent to that seen with statins [46]. Thus, vitamin C may have a beneficial effect on BP by mitigating the adverse effects of inflammation and oxidative stress. Specific functions of vitamin C have also been shown. These include effects on smooth muscle contractility of peripheral blood vessels and improvement of vasomotor dysfunction [47], prevention of nitric oxide inhibition of release of endothelium-derived relaxing factor [48], and prevention of free radical inhibition of prostacyclin synthetase. Ascorbic acid may also influence BP by maintaining the normal production and biological activity of nitric oxide through enhanced recycling of tetrahydrobiopterin, a cofactor that stabilizes and sustains the activity of endothelial nitric oxide synthase [49-52].

An important limitation of this study is the fact that the results depicted in Figures 1 and 2 are cross-sectional and therefore causality cannot be inferred. Also, the data represented in Figure 3 are limited by the fact that there were no plasma ascorbic acid measurements for the 9th annual visit, and therefore the association with change in BP, while longitudinal, is not truly prospective. In interpreting our findings it is important to consider the fact that dietary variables are often correlated with one another and may be correlated with lifestyle variables. Therefore, it is possible that plasma ascorbic acid concentration is simply a marker for another variable that influences BP. However, the association persisted even after controlling for BMI, race, education, and intake of fat, sodium and antioxidants. We also evaluated the effect of controlling for other potential confounders including smoking, alcohol consumption, television or video watching, and intake of total energy, calcium, and potassium, and none diminished the strength of the association.

Nevertheless, while we cannot confidently infer causality, our findings point to the important possibility that vitamin C may influence BP even among healthy young adults. This finding is of importance because lowering BP or attenuating increases in BP in young adults may lead to lower BP in older adults, which in turn may reduce the risk of age-associated vascular events. Therefore, our findings suggest that further study of the relationship between vitamin C and BP in this young-adult population is warranted.

## Conclusion

This is the first study to report on the relationship between plasma ascorbic acid concentration and BP in a cohort of young, normotensive women, aged 18–21 years. Plasma ascorbic acid concentration was found to be inversely associated with BP and with change in BP over the previous year. These inverse relationships were observed in a sample where two-thirds of participants were Black, a population that suffers disproportionately from elevated BP and its sequelae. Lowering BP or attenuating increases in BP in healthy young adults may lead to lower BP in older adults and reduced risk of age-associated vascular events. This study suggests that vitamin C may be an important factor in BP regulation even among health young adults, and that further study is warranted.

## Abbreviations

BP: blood pressurel CI: confidence interval; DASH: Dietary Approaches to Stop Hypertension; NHLBI: National Heart, Lung, and Blood Institute; BMI: body mass index

## Competing interests

The authors declare that they have no competing interests.

## Authors' contributions

GB and CDJ participated in data analysis and manuscript development. EPN conducted laboratory analyses and participated in manuscript editing. MH participated in data analysis and manuscript editing. PBC participated in the design of the study and manuscript editing. All authors have read and approved the final manuscript.
